# The Hepatitis C Virus Nonstructural Protein 2 (NS2): An Up-and-Coming Antiviral Drug Target

**DOI:** 10.3390/v2081635

**Published:** 2010-08-06

**Authors:** Ivo C. Lorenz

**Affiliations:** Laboratory of Virology and Infectious Disease, Center for the Study of Hepatitis C, The Rockefeller University, 1230 York Avenue, New York, NY 10065, USA

**Keywords:** nonstructural protein, dimer, protease, assembly, antiviral therapy

## Abstract

Infection with Hepatitis C Virus (HCV) continues to be a major global health problem. To overcome the limitations of current therapies using interferon-α in combination with ribavirin, there is a need to develop drugs that specifically block viral proteins. Highly efficient protease and polymerase inhibitors are currently undergoing clinical testing and will become available in the next few years. However, with resistance mutations emerging quickly, additional enzymatic activities or functions of HCV have to be targeted by novel compounds. One candidate molecule is the nonstructural protein 2 (NS2), which contains a proteolytic activity that is essential for viral RNA replication. In addition, NS2 is crucial for the assembly of progeny virions and modulates various cellular processes that interfere with viral replication. This review describes the functions of NS2 in the life cycle of HCV and highlights potential antiviral strategies involving NS2.

## 1. Introduction

About 3% of the world’s population are infected with Hepatitis C Virus (HCV). The existing treatment using PEGylated interferon-α in combination with ribavirin is efficient in only 55–60% of individuals infected with genotype 1 virus, and 80% of genotype 2 or 3 patients [1]. Moreover, the therapy is accompanied by serious side effects. This emphasizes the urgent need for specific antiviral drugs directed against key functions in the viral life cycle. Several compounds targeting HCV proteins or cellular functions involved in HCV replication are currently being tested clinically and will become available in the next few years (reviewed in [2]). However, mutant viruses that are resistant against these drugs have emerged *in vitro* and *in vivo*, suggesting that several enzymatic activities or other viral functions may have to be targeted in parallel in a combinatory approach, similar to the highly active antiretroviral therapy (HAART) against Human Immunodeficiency Virus (HIV) [3].

**Figure 1.**
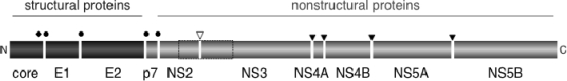
**Processing of the HCV Polyprotein.** The 3,011-amino acid polyprotein contains 10 HCV proteins, with the structural proteins (core, E1, E2) in the N-terminal third, and p7 and the nonstructural proteins (NS2, NS3, NS4A, NS4B, NS5A and NS5B) in the C-terminal two thirds. Cleavage of the polyprotein in the structural region and p7 occurs by action of the host cell signal peptidase (black circles) and the signal peptide peptidase (black diamond), whereas processing in the nonstructural region is mediated by the NS2-3 protease (open arrowhead) and the NS3/4A protease (closed arrowhead). The NS2-3 protease is shown as a dotted-line box. *N*, amino-terminus; *C*, carboxy-terminus.

The positive-strand RNA genome of HCV contains a single open reading frame encoding a 3,011-amino acid polyprotein precursor, which is co- and posttranslationally cleaved into individual proteins (Figure 1) [4]. The amino-terminus contains the structural proteins (core, envelope proteins E1 and E2), followed by a p7, a small transmembrane protein that assembles into a hexameric ion channel. The nonstructural (NS) proteins (NS2, NS3, NS4A, NS4B, NS5A and NS5B) reside in the carboxy-terminal two thirds of the polyprotein.

Processing into distinct proteins occurs by action of both host cellular and viral proteases (Figure 1). The signal peptidase (SPase) of the host cell cleaves at the junctions between core, E1, E2, p7 and NS2. In addition, cellular signal peptide peptidase (SPPase) liberates the core from its carboxy-terminal membrane anchor. Processing of the nonstructural region of the HCV polyprotein is mediated by two virus-encoded proteases: the NS3/4A protease, which cleaves all NS proteins downstream of NS3, whereas the NS2-3 protease mediates a single cleavage at the junction between NS2 and NS3.

The NS3/4A protease was characterized extensively by biochemical and biophysical methods, and structural information became available within a few years after the viral genome was isolated [5–10]. Therefore, NS3/4A was one of the first enzymes that was used in large antiviral drug screens against HCV. Two compounds targeting the NS3/4A protease, telaprevir and boceprevir, are expected to reach the market in the next few years [11–12]. This will be a significant improvement over the current treatment with PEGylated interferon-α in combination with ribavirin.

In contrast to NS3/4A, NS2 and the NS2-3 protease were studied by a few groups only, mainly due to the hydrophobic nature of NS2. However, significant progress has been made recently with the solution of the crystal structure of the NS2 protease domain, as well as the elucidation of multiple additional functions, many of which are crucial for maintaining important steps in the viral life cycle. With the help of these findings, NS2 has become an attractive candidate molecule for the development of antiviral drugs.

## 2. The NS2-3 Protease

NS2 (217 amino acids, molecular weight 23 kDa) is a nonstructural protein with a hydrophobic amino-terminal subdomain containing up to three putative transmembrane segments and a carboxy-terminal cytoplasmic domain. NS2 from the H77 strain (genotype 1a) is rapidly degraded in cells by the proteasome, whereas JFH1 NS2 (genotype 2a) is more stable [13–15]. These results indicate that the expression of NS2 is tightly regulated, and that protein turnover may vary between different HCV genotypes.

The C-terminal domain (residues 94–217) of NS2, together with residues 1–181 of NS3, forms the NS2-3 protease [16–21]. Cleavage at the NS2/NS3 junction is required for RNA replication of full-length HCV replicons [22], in the infectious tissue culture system [23], and in chimpanzees [24]. It is possible that not NS2 itself, but a free N-terminus of NS3 is necessary for the onset of replication. Whether NS2 has a direct role in the replication of HCV genomes remains to be determined.

Based on sequence alignments and mutagenesis experiments, a putative catalytic triad was identified shortly after the NS2-3 protease was discovered [16–17]: His 143, Glu 163 and Cys 184 are entirely conserved in all HCV isolates, and mutagenesis of any of these residues to alanine abrogates proteolytic activity. NS2-3 cleavage efficiency was increased by the addition of exogenous zinc and inhibited by metal chelators, indicating that NS2-3 might be a metalloprotease [17]. However, the dependence on zinc may be attributed to the NS3 portion of the NS2-3 protease, since the crystal structure of the NS3 protease domain contained a tightly coordinated zinc ion stabilizing the protein [8–9]. Limiting zinc may affect the folding of the NS3 protease domain, which in turn could interfere with NS2-3 processing [25].

The composition of the catalytic triad and comparison with other viral proteases led to the alternative hypothesis that NS2-3 was a cysteine protease [26]. This idea was further supported by the finding that classical cysteine protease inhibitors, such as N-ethylmaleimide or iodoacetamide, blocked NS2-3 cleavage [19–20].

The precise role of the NS3 protease domain in the processing of NS2-3 remains to be elucidated. Schregel *et al.* have shown that NS2 is a *bona fide* protease that shows basal activity with as little as two residues of NS3, although the presence of the entire protease domain (residues 1–181) significantly increased the cleavage efficiency [27]. They also ruled out a catalytic role for the zinc ion of NS3 in NS2-3 processing, supporting the classification of NS2-3 as a cysteine protease. It is possible that the NS3 protease domain aids the proper folding of NS2 by correctly placing the scissile bond at the NS2/NS3 junction in the active site.

### Crystal Structure of the NS2 Protease Domain

**Figure 2.**
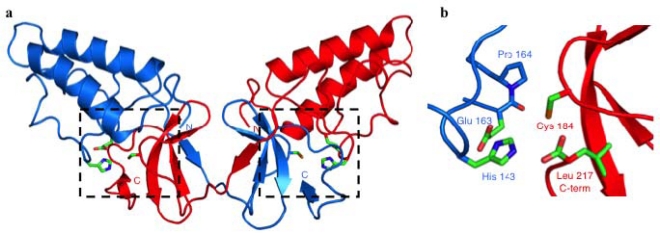
**Crystal Structure of the NS2 Protease Domain (NS2^pro^). (a)** NS2^pro^ forms a dimer with an N-terminal alpha-helical subdomain and a domain-swapped C-terminal antiparallel beta sheet. The two dashed boxes indicate the active sites, with the catalytic triad represented in green. **(b)** Detailed view of the NS2^pro^ active site. His 143 and Glu 163 originate from one monomer, and the nucleophilic Cys 184 is provided by the other monomer. The C-terminal Leu 217 remains bound in the active site. Pro 164, which lies immediately downstream of the catalytic Glu residue, is in a *cis*-peptide conformation. *N*, amino-terminus; *C*, carboxy-terminus. This figure was generated using PyMOL (http://www.pymol.org).

Confirmation that NS2-3 was a cysteine protease came with the solution of the crystal structure of the NS2 portion of the NS2-3 protease (Figure 2a) [28]. NS2^pro^ (residues 94–217) showed a novel overall fold with no known cellular or viral structural homologues. One solvent-exposed face of the structure was hydrophobic, which has led to the hypothesis that the protease domain may be peripherally inserted into a cellular membrane. The crystal structure showed that NS2^pro^ formed dimers, with extensive contacts between the two molecules and a domain exchange of the carboxy-terminal subdomains.

His 143, Glu 163 and Cys 184, which were identified earlier as putative active site residues, formed a catalytic triad that could be superimposed with other viral cysteine proteases, thus firmly establishing the classification of NS2 as a cysteine protease (Figure 2b). Interestingly, the NS2^pro^ dimer contained a pair of composite active sites, with His 143 and Glu 163 contributed by one monomer, whereas Cys 184 originated from the other monomer. This implied that dimerization of NS2 was required for processing at the NS2/NS3 junction. It was suggested previously that the NS2-3 cleavage reaction might be bimolecular [19,29], and the dimeric enzymatic mechanism proposed based on the crystal structure of NS2^pro^ was confirmed by a series of experiments in mammalian cells [28].

The unusual arrangement of the NS2^pro^ active site offers interesting regulating possibilities. The finding that NS2 dimerization is required for proteolysis suggests that certain concentrations of the protein have to be accumulated before NS2-3 processing and thus initiation of RNA replication can occur. A delayed enzyme kinetic may allow the virus to generate sufficient amounts of NS3/4A to antagonize the activation of the interferon pathway before the onset of viral replication [30].

The crystal structure also showed that the backbone of the carboxy-terminal residue of NS2, Leu 217, remained bound in the active site, making contact to the residues of the catalytic triad. This suggests that Leu 217 may prevent other substrates from accessing the active site of NS2^pro^, locking the enzyme in an inactive conformation. This may represent an additional mechanism of the virus to tightly regulate processing of NS2-3.

Pro 164, which follows immediately downstream of the catalytic residue Glu 163, showed a *cis*-conformation in the crystal structure. The *cis*-peptide configuration of Pro 164 may be required to establish the correct geometry at the active site of NS2^pro^, or for the molecule to form dimers. The presence of a *cis*-proline implies that a cellular enzyme, a prolyl-peptidyl *cis-trans* isomerase, is required to mediate proper folding of NS2.

## 3. Role of NS2 in Virus Assembly

For many years, the protease activity was the only function known for NS2. In recent years, NS2 was shown to be involved in a variety of other processes during the viral life cycle, including a crucial function in virus assembly. Using deletion mutants in bicistronic HCV RNAs containing an internal ribosomal entry site (IRES) from encephalomyocarditis virus (EMCV) between NS2 and NS3, several groups showed that full-length NS2 was required for production of virus particles [23,31]. Mutating residues of the catalytic triad to alanine did not impair virus production, indicating that the NS2 protease activity itself was not required to generate progeny virions.

The N-terminal transmembrane segment of NS2 seems to play an important role in HCV particle production. Experiments with chimeric HCV derived from two different genotypes or two strains of the same genotype showed that virus production was most efficient when the transition point between the two genomes was placed after the first transmembrane segment of NS2 [32]. These results indicate that the N-terminus of NS2 may interact with upstream structural proteins via its first transmembrane segment, whereas the C-terminal part of NS2, including the protease domain, binds to downstream nonstructural proteins.

The structure of the first transmembrane segment of NS2 was solved by solid-state NMR, showing a flexible helix in the N-terminal part (residues 3-11) connected to a stable alpha helix (residues 12–21) via two glycine residues [31]. The amino acid residues on one face of the helix are highly conserved within the same HCV genotype, and mutagenesis of residues in this region significantly reduces virus production. Gly 10, a residue that is entirely conserved amongst all HCV isolates, may act as a “hinge” to enable intramembrane protein-protein interactions.

Yi *et al*. described a role for NS2 at a late stage of virus assembly, subsequent to the involvement of core, NS3 and NS5A [15]. In addition, they showed by confocal microscopy experiments that NS2 co-localized with several other HCV proteins, especially E2 and NS5A, indicating that NS2 may directly or indirectly interact with various other HCV structural and nonstructural proteins. The authors suggested that similar to NS2A in the flaviviruses [33], HCV NS2 may induce membrane alterations and envelope protein rearrangements to facilitate virus assembly and release.

These findings demonstrate that multiple regions of NS2, including the N-terminal transmembrane segment and the cytoplasmic protease domain, are essential for virus production. NS2 may act as a “bridging factor” to bring core, E1/E2 and the viral RNA, which is synthesized by the replication complexes composed of nonstructural proteins, into close proximity for incorporation into virions. The detailed molecular mechanisms of these processes remain to be elucidated in future studies.

## 4. Cellular Proteins Interacting with NS2

Various interactions between NS2 and cellular proteins have been reported. NS2 was suggested to interfere with apoptosis [34], inhibit cell proliferation by inducing cell cycle arrest [35], and to regulate cAMP-dependent pathways [36], cytokine expression [37] and liver fat metabolism [38]. Moreover, NS2 is a target for phosphorylation by casein kinase 2 (CK2) and subsequent degradation [13]. Since most of these experiments were carried out in mammalian tissue culture systems using overexpressed NS2, the *in vivo* relevance of some these functions remains to be determined.

In addition, cellular proteins that regulate the synthesis and folding of NS2 were described. The ATP-hydrolyzing activity of the molecular chaperone Hsp90 was shown to be necessary for NS2-3 cleavage in cell-free assays and in tissue culture experiments [39]. The chaperone may be required for the proper folding of NS2, or for the correct spatial arrangement of the NS2 and NS3 domains to allow cleavage of the scissile bond.

Cyclosporine A has been reported to decrease the replication levels of HCV genomes *in vitro* and *in vivo* by many groups (reviewed in [40]). Recently, it was revealed that NS2 was one of the enzymes targeted by cyclosporine A [41]. The inhibitory effect is mediated by the host cellular peptidyl-prolyl *cis-trans* isomerase Cyclophilin A. The most likely target proline in NS2 is Pro 164, which is highly conserved in all HCV genotypes. Cyclophilin A would promote isomerization of Pro 164 from a *trans-*to a *cis*-conformation, allowing proper folding of the enzyme and establishing the correct geometry at the active site for NS2-3 cleavage to occur (Figure 2b).

## 5. Potential Antiviral Therapies Targeting NS2

In the past few years, significant progress has been made on the biochemical and biophysical characterization of HCV NS2, and multiple roles of the protein in the viral life cycle have been described. The proteolytic activity of NS2-3 is required for the onset of RNA replication, and the protein has additional important roles in virus assembly as well as in the modulation of the host cell response to viral infection. These studies demonstrate that NS2 is a key enzyme for many functions that are essential for the viral life cycle, making it an attractive target for antiviral therapies. The following sections describe possible intervention strategies that involve NS2 to limit HCV replication and spread.

### 5.1. Protease Active Site

Inhibiting the enzymatic activity of a viral protease is an effective way to interfere with replication of the virus, as demonstrated for the new HCV NS3/4A inhibitors (see above), as well as for the HIV protease inhibitors, which are an integral component of HAART (reviewed in [42]). For many years, the HCV NS2-3 protease was not considered a good target for antivirals because cleavage at the NS2/NS3 junction was thought to be a unimolecular reaction *in cis*. Autoproteolytic processing at the C-terminus of NS2 would imply that processing kinetics may be too fast for inhibition by a drug. The finding that NS2-3 is a dimeric enzyme with two active sites that are each composed of residues from both monomers significantly prolongs the ‘window of opportunity’ to block proteolysis with small compounds.

Although the crystal structure of NS2^pro^ represents the post-cleavage form of the NS2-3 protease, the spatial arrangement of the catalytic triad of NS2 is similar to other viral cysteine proteases. However, the protease inhibitor profile of NS2-3 was ambiguous, since certain classic cysteine protease inhibitors, such as E-64, had no effect on NS2-3 processing [20]. On the other hand, serine protease inhibitors, including tosyl phenyl chloromethyl ketone (TPCK) and tosyl L-lysine chloromethyl ketone (TLCK), inhibited NS2-3 cleavage [19–20]. This finding may be explained by the fact that NS2 has an active site geometry that is similar to serine proteases, analogous to cysteine proteases from other virus families, e.g. the picornaviral 3C proteases [43]. This unusual feature may help in designing and modeling small compounds that specifically target the active site of NS2.

In addition to small molecule inhibitors, NS2-3 processing can be inhibited by peptides derived from NS4A [20,44]. NS4A binds to the N-terminus of NS3 and acts as a cofactor for the NS3 protease. NS4A peptides thus probably interfere with proper placement of the scissile bond at the junction between NS2 and NS3 in the protease active site. Consistent with the finding that the C-terminal Leu 217 remains bound in the active site of NS2^pro^, peptides derived from the C-terminus of NS2 also inhibited NS2-3 proteolysis, although to a lesser extent [20]. Therefore, peptides derived from various regions of the HCV polyprotein may be used to prevent NS2-3 cleavage.

### 5.2. NS2 Dimerization

Experiments with transfected NS2-3 or full-length HCV genomes in mammalian cells suggested that dimerization of NS2-3 was required for proteolytic activity and therefore initiation of replication [28]. Moreover, dimerization of NS2 is also important for another function aside from proteolysis, as shown by the analysis of NS2 mutants in the context of bicistronic HCV genomes with an EMCV IRES inserted between NS2 and NS3 [45]. While replication was unaltered, virus production was significantly lower for certain mutations at the dimer interface of NS2, whereas amino acid changes at the protease active site had no effect. Although it cannot be ruled out completely that these mutations affect the folding process of the molecule, these findings suggest that dimerization is crucial for multiple functions of NS2.

In the crystal structure of NS2^pro^, the total buried surface area between the two subunits of a dimer is 1300 Å^2^, and most residues that make contact between the two molecules are highly conserved [28]. Based on the crystal structure, small compounds or peptides that prevent NS2 dimerization could be designed and modeled *in silico* by focusing on areas of an NS2 molecule that are involved in contacting the other monomer.

Targeting the dimerization interface of a protein to interfere with its function(s) is an emerging concept to develop therapeutic drugs [46]. It was successfully applied for multiple HIV enzymes, including the protease, the integrase, and the two subunits of the HIV reverse transcriptase (reviewed in [47]). A similar strategy was suggested for inhibiting chemokine receptors, many of which require dimerization to trigger intracellular signaling cascades [48].

### 5.3. Functions of NS2 Involved in Virus Assembly

Recent findings from experiments with the infectious HCV cell culture system clearly suggest that NS2 plays an important role in virus assembly. Therefore, the regions of NS2 that participate in the generation and release of viral particles can be targeted by antiviral drugs. Several residues in the NS2 protease domain that did not affect RNA replication, but impaired release of infectious virus, were identified [14–15,45]. However, the precise role of these residues in the assembly process, and their interactions with other viral and cellular factors involved in virus particle production, may have to be determined before compounds that specifically interfere with virus assembly can be developed.

### 5.4. Interactions between NS2 and other Viral Proteins

Biochemical experiments [49–50] as well as forward genetic selection in cell culture adaptation experiments after mutational analysis [15,51–53] have shown that NS2 is involved in a complex network of interactions with other viral proteins. Although the precise sites of interaction between NS2 and other proteins has not been mapped, and some of the interactions may be indirect, inhibitors could target the interaction of NS2 with other viral proteins, especially NS3. This strategy may include the design of compounds that bind to the surface of NS2 in conserved regions, which are likely to be involved in interactions between NS2 and other viral (or cellular) proteins.

### 5.5. Cellular Proteins Interacting with NS2

Aside from targeting NS2 directly, antiviral therapies could also be directed at cellular proteins interacting with NS2. Inhibiting host cell factors has a significant advantage over blocking viral enzymes, since host cell functions are much less likely to develop resistance mutations. On the other hand, a cellular enzyme probably has crucial functions in the host organism. Therefore, the antiviral therapy may have detrimental side effects unless there is a redundant mechanism for that cellular function. However, one drug targeting a cellular enzyme might be effective against multiple viruses, which often rely on the same host cell functions for their replication.

Various drugs that target cellular functions have been implied in inhibiting HCV replication. As mentioned above, cyclosporine A was shown to decrease the proteolytic activity of NS2 by blocking the cellular peptidyl-prolyl *cis-trans* isomerase Cyclophilin A [41]. Several compounds derived from cyclosporine A have shown promising results in treating HCV infection in clinical trials. Screening for compounds that target other host cell factors interacting with NS2, e.g. the Hsp90 heat shock chaperone or casein kinase 2, may lead to the identification of additional inhibitors of HCV replication.

## 6. Conclusions

With almost half of the individuals infected with genotype 1 HCV not responding to the current treatment with interferon-α and ribavirin, novel highly efficient therapies are sorely needed. A new generation of HCV-specific compounds, most of which target either the NS3 protease or the NS5B RNA-dependent RNA polymerase, is expected to become available soon. However, viral escape mutants that lead to resistance against these drugs have already emerged. For an efficient and sustainable therapy against HCV infection, a strategy targeting multiple enzymatic activities or other functions of the viral proteins has the best chance for success. Recent advances in elucidating the structure and function of NS2 emphasized on the crucial role of this protein in the HCV life cycle. Therefore, NS2 is an excellent candidate molecule to develop additional antiviral therapies against HCV infection.
